# Inducible T-cell co-stimulators regulate the proliferation and invasion of human hepatocellular carcinoma HepG2 cells

**DOI:** 10.1186/s40659-017-0150-7

**Published:** 2018-01-09

**Authors:** Yaning Wei, Yanan Wang, Aimin Zang, Yanhong Shang, Zizheng Song, Zhiyu Wang, Yangyang Wang, Hua Yang

**Affiliations:** 1 Departments of Oncology, Affiliated Hospital of Hebei University, Baoding, 071000 China; 2 Department of Pathology, Affiliated Hospital of Hebei University, Baoding, 071000 China; 3 Department of Gastroenterology, Affiliated Hospital of Hebei University, Baoding, 071000 China

**Keywords:** Inducible T-cell co-stimulator (ICOS), Liver cancer, Proliferation, Invasion, PI3K/AKT

## Abstract

**Background:**

This study determined the regulatory effects of inducible T-cell co-stimulators (ICOS) in human hepatocellular carcinoma HepG2 cells using a RNA interference (RNAi) technique.

**Methods:**

A RNAi technique was used to knockdown the expression of ICOS. ICOS expression after knockdown was detected as mRNA and protein levels by RT-PCR and Western blot, respectively. A MTT colorimetric assay was used to detect cell proliferation, and the Transwell assay was used to detect cell invasion. Western blot was carried out to detect the level of Bcl-2, AKT, and PI3K protein expression in different groups.

**Results:**

The proliferation of HepG2 cells were significantly decreased after ICOS siRNA transfection (EG group). Similarly, the results of the Transwell experiment showed that invasion of HepG2 cells in the EG group was clearly reduced compared to the negative control (NC) and blank control groups (CON). Western blot analysis showed that knockdown of ICOS expression reduced the levels of Bcl-2 and AKT, and also significantly up-regulated the level of PI3K phosphorylation (P < 0.01).

**Conclusion:**

Down-regulating ICOS expression in HepG2 cells suppressed cell proliferation and invasion. The underlying mechanism may be related to the expression of the downstream factor, PI3K/AKT.

## Background

Primary hepatocellular carcinoma (HCC) is a malignant tumor with a high incidence and mortality rate in China. The incidence of HCC is 28.7/100,000 in China, and the morbidity rate exceeds one-half of the global incidence. The incidence of HCC ranks 4th among malignant tumors in China [1, 2]. Currently, large-dose cytotoxic chemotherapy and surgical excision can improve the prognosis to some extent; however, a clear and thorough understanding of the pathogenesis of HCC is still lacking. Indeed, the probability of metastasis and recurrence of HCC is at a high level, thus further studies involving the genes which significantly affect HCC as well as the pathogenesis are warranted [1–5].

In humans the generation and maintenance of antigen-specific T lymphocyte-mediated immune responses need two signals (specific antigen signals provided by the compatible composite-peptide of main tissues and co-stimulatory signals provided by antigen-presenting cell surface molecules). In 1999, scientists found a new co-stimulatory molecule in the human immune system [inducible co-stimulators (ICOS)]. ICOS are related to T cells [6–8]. Recent studies have shown that ICOS may have certain functions involving the proliferation and invasion of tumors [7, 8]. Tumor cells can escape from immune system surveillance via several mechanisms, and further grow, divide, and proliferate. Recent studies have shown that T cell-mediated immunity is a major anti-tumor immune mechanism in humans, and the activation of initial T cells only act under the participation of co-stimulatory molecules. Thus, co-stimulatory signals may play an important role in the control of tumor cells [6–12]. Recently, Sanmamed et al. [11] reported that the co-stimulatory molecule, the ICOS gene, may serve as a target for tumor treatment. Studies regarding ICOS in liver cancer, however, are far from sufficient, and the literature related to cell or animal experiments to date have limited our further understanding of the pathogenesis of liver cancer. HepG2 cells were used in the current study with RNAi technology to knockdown the expression of the ICOS gene of co-stimulatory molecules in hepatoma cells, and to analyze the cell proliferation and invasion capacities of HepG2 cells after ICOS gene knockdown. The present study provides the experimental and theoretical bases for exploring the effect of the ICOS gene in liver cancer and also provide a new scientific perspective to illustrate the pathogenesis of liver cancer.

## Methods

### Cell line and reagents

The related reagents are described below. DMEM cell culture medium was purchased from Gibco Company (Waltham, Massachusetts, USA). Trypsin was purchased from Sigma Company (St. Louis, Missouri, USA). Lipidosome LIPOFECTAMINE 2000, Opti-MEM low-serum medium, rabbit-anti-human polyclonal antibody, rabbit-anti-rat polyclonal antibody marked with HRP, and siRNA for the negative control group were purchased from Invitrogen Company (Waltham, Massachusetts, USA). Protein lysis buffer (RIPA) was purchased from Novogen Company (Mauguerand, France). A protein quantitative reagent (BCA kit) was purchased from Pierce Company (Waltham, Massachusetts, USA). MTT and BCA staining kits were purchased from Ribo Bio. Co., Ltd. (Beijing, China). The HCC cell line, HepG2, was provided by the Jiangsu Key Laboratory of Medical Molecular Technology (Jiangsu, China).

### HepG2 cell cultures

HepG2 cells were removed from liquid nitrogen, and quickly thawed in a water bath at 37 °C. After centrifugation, the cells were collected, and placed in DMEM culture medium containing 10% fetal calf serum, then cultured at 37 °C in a 5% CO_2_ incubator. The culture medium was replaced periodically. After the tumor cells grew to approximately 80% of the bottle wall, they were digested and transferred by 0.25% trypsin, then cultured in a fresh culture bottle.

### Design and synthesis of siRNA

The ICOS gene sequences were obtained from the Genebank database, and the corresponding siRNA sequences for the ICOS gene were designed using siRNA design software. The sequences were as follows: positive-sense strand, 5′-GGAACUUGCCAUCAAGAUCTT-3′; and negative-sense strand, 5′-AAUGUCGAUAGGAACUUGCTT-3′. The sequences for siRNA in the negative control group were as follows: positive-sense strand, 5′-CCAACUUTCCAUCAACAUCTT-3′; and negative-sense strand, 5′-GGUCUAGAUACCTTCUUGGAA-3′.

### Grouping and transfection

The experiment was divided into the following three groups: ICOS siRNA transfection group (Group A); negative control (NC) group (transfection with an irrelevant siRNA sequence; Group B); and blank control (CON) group (Group C). HepG2 cells (1 × 10^5^ cells/mL) were cultured in a 6-well plate in a 5% CO_2_ incubator for siRNA transfection. The cells were washed 2 times in serum-free DMEM, then added to serum- and antibiotic-free DMEM culture medium (0.8 mL/well), and finally cultured at 37 °C in a 5% CO_2_ incubator for 1 h. Then, 10 μL of siRNA solution and 175 μL of serum-free DMEM were added and mixed uniformly so that the siRNA transfection solution was acquired. After 6 h of cell and siRNA transfection solution co-culturing, the transfection rate was observed under a microscope in bright and fluorescent field.

### Detection of the ICOS mRNA expression by RT-PCR

To detect the level of ICOS mRNA in the transfected cells, HepG2 cells were collected and the total RNA was extracted according to the instructions in the kit provided by Alphainnotech Company (city, state, USA). The ratio of the absorption value at 260–280 nm was measured on a micro-spectrophotometer to evaluate the purity of RNA; the ratio was 1.8 ~ 2.1. Primer 5.0 was adopted for primer design (Ribo Bio. Co. Ltd.). The primers for ICOS detection were as follows: forward primer, 5′- CGT CAC GAC CTA CGA TA-3′; and reverse primer, 5′- GCC CCG CGC CGA GGC AG-3′. *β*-actin was used as the internal reference; the primers were as follows: forward primer, 5′- GGT GTG ATGGTG GGT ATG GGT-3′; and reverse primer, 5′- CTG GGT CATCTT TTC ACG GTC-3′. Amplification was performed using a Takara reverse transcription and amplification kit (Tokyo, Japan). The relative expression of mRNA in all samples were calculated according to 2^−ΔΔCt^ method.

### Detection of ICOS, AKT, Bcl-2, and PI3K protein expression by western blot

Cell lysis buffer (Wuhan Zhongzhi Biotechnologies Co., Ltd., Wuhan, China) was used to split the HepG2 cells into different groups for total protein extraction from cells. Phosphatase inhibitors (Sigma Company) were added into the corresponding cell lysis buffers to detect the level of protein phosphorylation. SDS-PAGE electrophoresis was performed after the extracted total protein in different groups was denatured. After electrophoresis, the protein was transferred to prepared nitrocellulose membranes, and the nitrocellulose membranes were sealed with 5% skim milk powder for 2 h. After washing, the first antibody was added and incubated overnight at 4 °C. On the next day, the membranes were washed once in PBS and twice in TBST. Then, the second antibody labeled with horseradish peroxidase was added and incubated at room temperature for 2 h. After the membrane was washed, ECL chemiluminescence was used for the signaling exposure with X-ray film, then fixed and scanned. *β*-actin was used as the internal reference. The dilution ratios of antibodies for anti-ICOS, -AKT, -Bcl-2, and -*β*-actin or the corresponding phosphorylation antibodies were 1:1000 (Santa Cruz Biotechnology, Inc., Santa Cruz, CA, USA), and the second goat anti-rat or goat anti-rabbit antibodies labeled with HRP were diluted to 1:5000.

### Detection of HepG2 cell proliferation by the MTT colorimetric method

HepG2 cells (1 × 10^5^ cells/mL) were incubated in 96-well plates. After siRNA transfection for 6 h, 20 μL of MTT was added to each well periodically and incubated at 37 °C for 4 h. The liquid supernatant in the well was removed, then 150 µL of DMSO was added to dissolve the crystal. An enzyme-linked immune detector (Alphainnotech Company) was used to detect the absorbance value for each well at 570 nm.

### Transwell invasion experiments

For the Transwell assay, Matrigel was thawed at 4 °C overnight, then diluted (5–1 mg/mL) in cold serum-free DMEM cell culture medium. Diluted Matrigel (100 μL) was placed in the upper chamber (polycarbonate membrane [diameter, 8 μm]) of a 24-well plate. The Matrigel was incubated in the Transwell chamber at 37 °C for at least 4 h to gel. HepG2 cells were harvested and suspended in serum-free DMEM medium at a density of 10 × 6 cells/mL. The gelled Matrigel was gently washed with warmed serum-free DMEM culture medium. The cell suspension (100 μL) was placed onto the Matrigel and 700 μL of DMEM culture medium containing 10% fetal calf serum was added to the lower Transwell chamber, and incubated at 37 °C for 18 h. The Transwell chambers were removed from the 24-well plates, twice-washed in pre-cooled PBS, fixed with 4% paraformaldehyde, and stained with 0.1% crystal violet for 5–10 min. After staining, the cell suspensions were photographed under a microscope (Olympus Company, Japan), and the number of cells passing through the Transwell membranes in different groups was counted. The experiments were repeated 3 times to acquire the average.

### Statistics processing

Quantitative data are expressed as the mean ± standard deviation ($\overline{x} \pm s$), and SPSS 23.0 software was used for statistical analysis. The measurement data between two groups were compared with a t-test, and the measurement data among several groups were compared with a one-way analysis of variance (ANOVA). Fisher’s LSD test was used to calculate statistical significance. A P < 0.05 indicates a statistically significant difference.

## Results

### Detection of the siRNA transfection rate

**Fig. 1:**
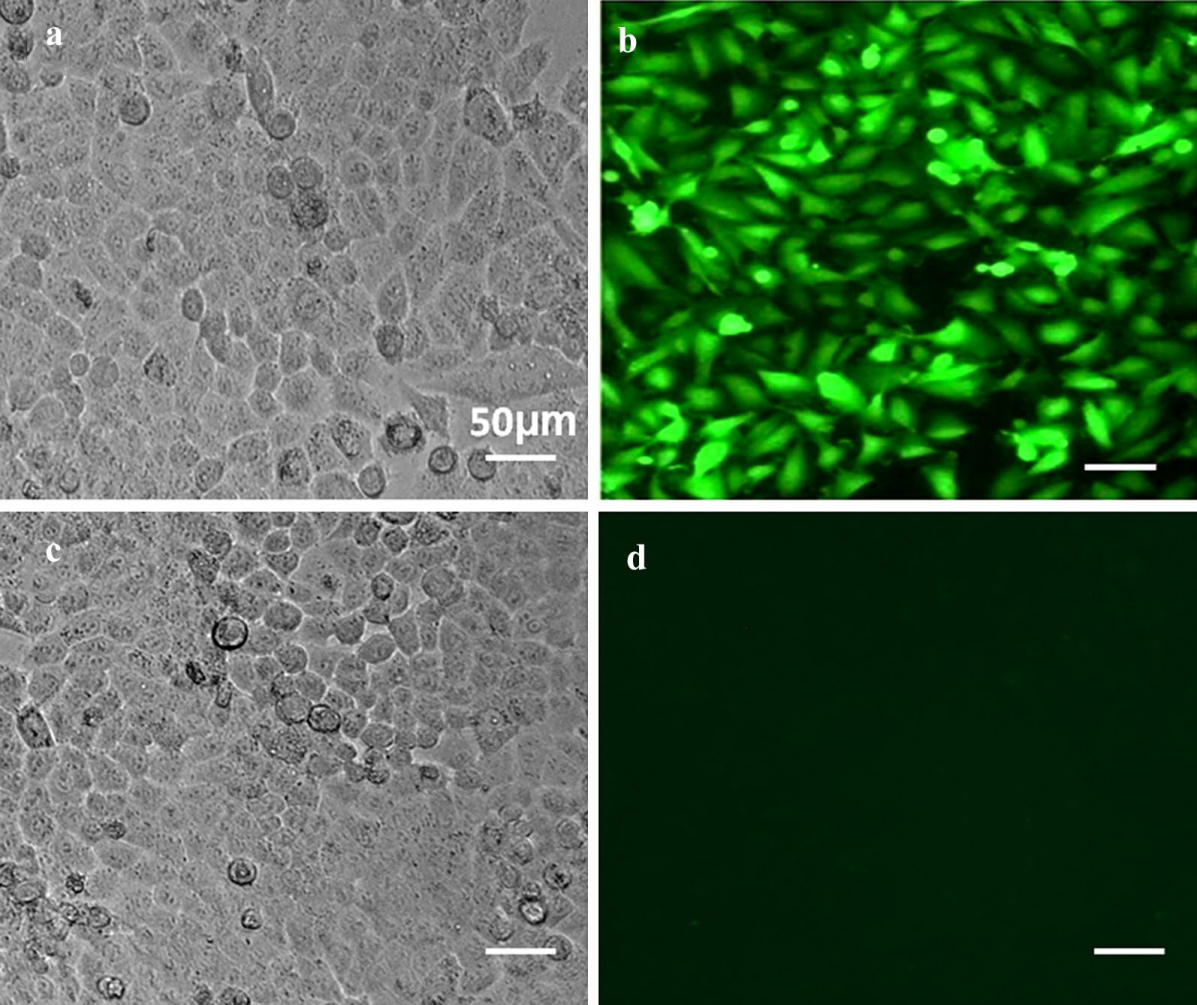
Detection results of siRNA transfection rate (×100). **a** Negative transfection control observed under the fluorescence microscope. **b** Positive transfection observed under the fluorescence microscope; **c**, **d** Negative transfection control observations under the bright field and fluorescence microscope with the same visual field

After transfection for 6 h, the cells were observed under a fluorescent and bright field microscope to determine the transfection rate. Figure 1a shows the negative transfection control. Figure 1b shows the positive transfection of > 70% compared with the negative control.

### Expression of ICOS mRNA and protein levels after ICOS siRNA transfection

**Fig. 2:**
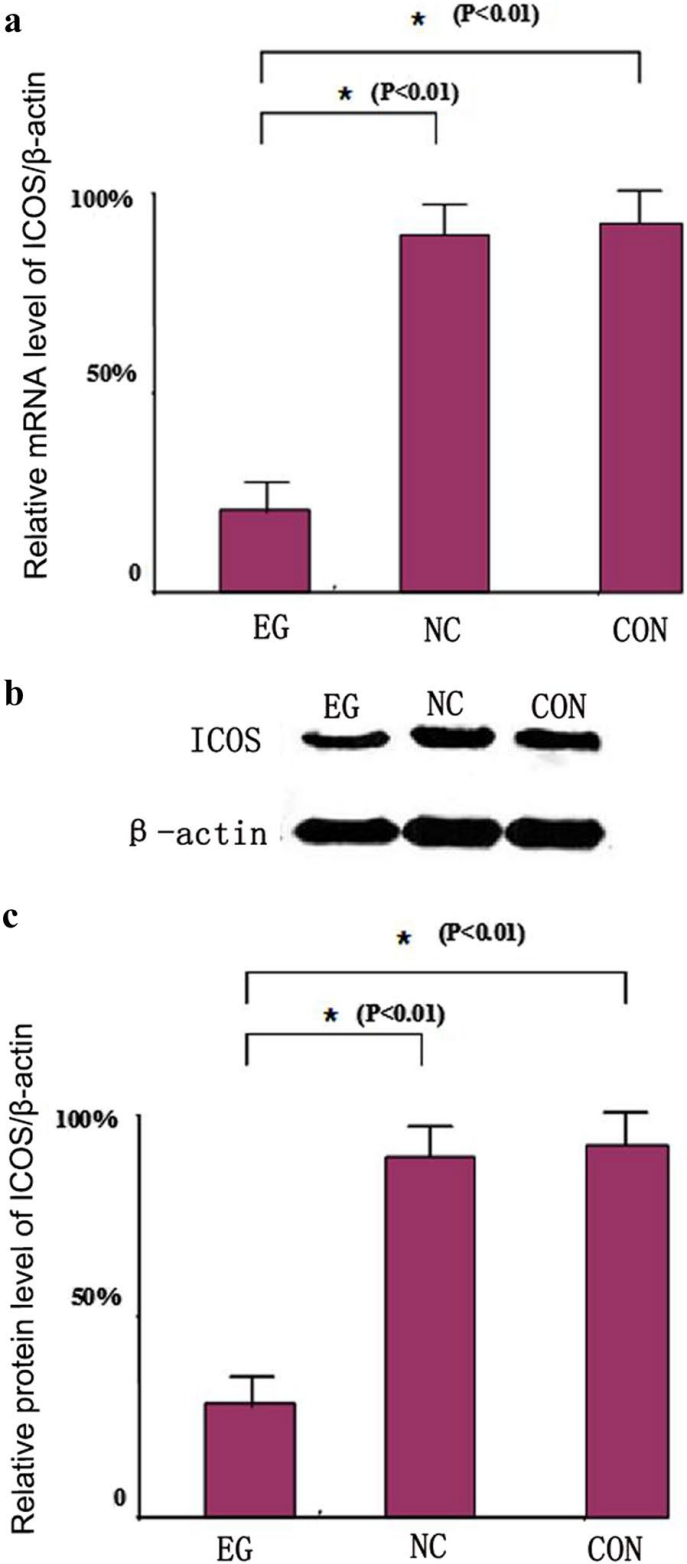
Detection results of levels of ICOS in mRNA and protein expression after siRNA transfection. **a** RT-PCR results of ICOS-mRNA after transfection; **b** western blot results of ICOS protein expression after transfection; **c** histogram of three independent repeated test results, in which EG represents the results of the experimental group, NC represents the results of the negative control group, and CON represents the results of the blank control group; *P < 0.01

To further clarify the influence of transfection on the targeted gene and protein, we determined the expression of ICOS mRNA and protein levels in the different groups. As shown in Fig. 2a, the results of RT-PCR indicate that after ICOS siRNA transfection (EG), the mRNA expression of ICOS in the EG group was significantly reduced compared with the NC and CON groups. Thus, the ICOS siRNA expression carrier effectively transfected cells and successfully reduced the expression of ICOS mRNA.

In addition, we determined the expression of ICOS protein in different groups by Western blot. As shown in Fig. 2b, compared with the NC and CON groups, the expression of ICOS protein in the EG group was significantly reduced, indicating that the expression of ICOS protein in HepG2 cells was successfully reduced through the expression carrier of ICOS siRNA. The expression of ICOS protein was quantitatively analyzed using three independently repeated experiments, and the inhibition efficiency of ICOS protein was calculated after ICOS siRNA transfection to be approximately 65%, as shown in Fig. 2c.

### Influence of ICOS knockdown on the proliferation and infiltration of HepG2 cells

**Fig. 3:**
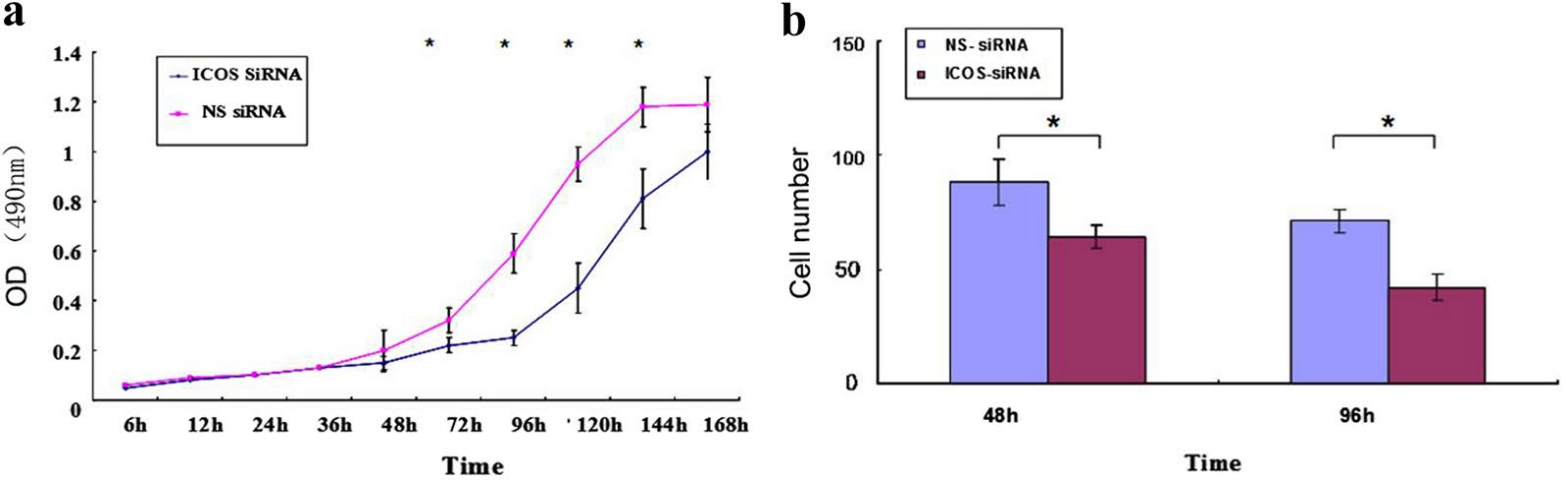
Influence of ICOS knockdown on the proliferation and infiltration of HepG2 cells. **a** Growth curve of HepG2 cells after transfection; **b** influence of transfection on the infiltration of HepG2 cells; *P < 0.01. The experiment was independently repeated three times

The proliferation of cells in different groups was measured by detecting the OD values of HepG2 cells 0, 6, 12, 24, 36, 48, 72, 96, 120, 144, and 168 h after transfection using the MTT experiment method. Then, the cell growth curve was plotted for all groups as time (horizontal axis) versus average absorbance values of A570 (vertical axis) in all groups (Fig. 3a). Statistical methods were used for data analysis. ICOS knockdown significantly suppressed the proliferation of HepG2 cells, and the effect was more pronounced as the time after transfection was prolonged (*P < 0.05).

To detect the influence of ICOS knockdown on the infiltration of HepG2 cells, Transwell invasion assays were performed. As shown in Fig. 3b, compared with the results in the NC and CON groups, the number of invaded chambers in the EG group was significantly reduced (P < 0.05). Therefore, invasion of HepG2 cells was suppressed by reducing the expression of ICOS.

### Influence of ICOS knockdown on the expression of AKT and Bcl-2 protein, and phosphorylation of PI3K in HepG2 cells

**Fig. 4:**
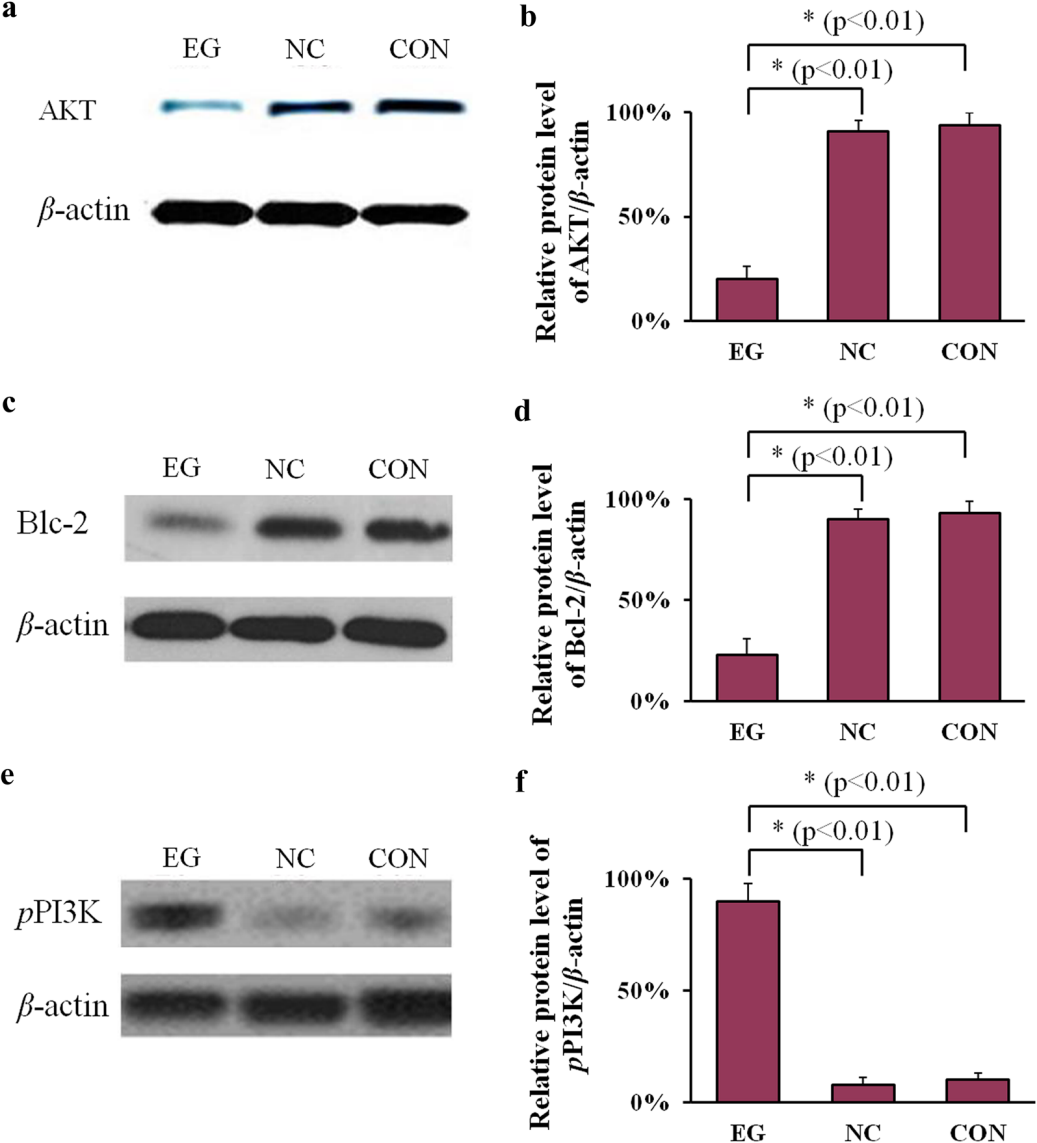
Expression of AKT, Bcl-2, and *p*PI3K in HepG2 cells after transfection. **a** Expression of protein AKT in different groups; **b** expression of protein AKT in three independently repeated tests; **c** expression of protein Bcl-2 in different groups; **d** expression of Bcl-2 protein in three independently repeated tests; **e** expression of *p*PI3K protein in different groups; **f** expression of *p*PI3K protein in three independently repeated tests; *P < 0.01

AKT, also known as protein kinase B, is an important kinase downstream of ICOS. AKT plays an important role in the generation and development of liver cancer cells. Therefore, the expression of AKT was determined in the different groups. As shown in Fig. 4a, compared with the NC and CON groups, the expression of AKT protein in the EG group was significantly reduced (n = 3, P < 0.05), as shown in Fig. 4b.

Bcl-2 is an important biological marker of cell apoptosis. Western blot was carried out and the results are shown in Fig. 4c. Compared with the results of NC and CON groups, the expression of Bcl-2 in the EG group was significantly reduced (n = 3, P < 0.05), as shown in Fig. 4d. ICOS knockdown suppressed the expression of Bcl-2, and thus may suppress HepG2 cell apoptosis.

Because phosphorylation of PI3K protein plays an important role in the ICOS signal channel, Western blot was performed to detect the expression of PI3K phosphorylation. As shown in Fig. 4e, compared with the NC and CON groups, PI3K phosphorylation in the EG group was significantly enhanced, as shown in Fig. 4f.

## Discussion

Liver cancer is a common malignant tumor. According to the data issued by the World Health Organization (WHO), the global incidence of primary HCC is increasing year-after-year, and > 600, 000 new cases are reported each year, ranking 5th among all malignant tumors. Moreover, the liver cancer-related fatality rate ranks 3rd [1, 2]. In addition, chemotherapy and radiation therapy do not have good specificity for liver cancer cells, and may also bring some toxic or side effects on the body and cannot to be tolerated, which will lead to a poor curative effect and prognosis of HCC, short overall survival time, and high recurrence and case fatality rates [13, 14].

RNA interference is a gene silencing technology which can induce base sequence homology mRNA degradation with the use of double-stranded RNA (dsRNA) to reduce the expression of the targeted gene. A gene delivery system is the key to gene therapy and RNA interference technology [4–8]. With the development of molecular biology and immunology, many molecules that are associated with the biologic characteristics of tumor have good application prospects. Therefore, studying the molecules associated with tumor biology function has great significance [7, 8].

ICOS, an important co-stimulatory molecule, participates in a variety of physiologic functions, such as cell inflammation and immune response [9]. ICOS expression can be detected in a wide variety of tumor tissues, such as melanomas, lungs, ovaries, and colon, indicating that IOCS expression is correlated with the development and prognosis of several tumors [10–12]. The ICOS receptor is a member of the B7 family and is expressed on the tumor cell surface; the ICOS receptor plays an important role in tumor immunity. The expression of ICOS and its receptor was determined in blood tumor cell lines, and the results showed that both are highly expressed in FBL3, A20, and P388 cells, i.e., ICOS and its receptor are closely associated with hematologic tumors [15–18]. Other studies also showed that cytokine IL-4 and IL-10 expression is reduced and the tumor immune response is decreased by inhibiting the expression of ICOS and its receptor, which is not conducive to activation of T cells [19]. In spite of this, immunologists, both domestically and internationally, are of the opinion that immune function of ICOS on tumors can be induced in NK cells, and the survival rate of melanoma cells is closely associated with the expression of ICOS [20]. Research regarding the correlation between ICOS and HCC has not been conducted to date, and no corresponding in vitro cell and in vivo animal experiments have been conducted [21].

In recent years, as progress is made towards elucidation of tumor molecular mechanisms, many scientists think that the occurrence and progress of cancer may be related to tumor cell immune evasion strategies [22, 23]. Therefore, RNAi was used in the current study to investigate the biological characteristics of HCC HepG2 cells under suppression of ICOS. The results confirmed that the reduction in ICOS expression significantly inhibited the proliferation and invasion of HCC HepG2 cells, and indicate that ICOS participates in the regulation of proliferation and the attack process of human HCC HepG2 cells, which plays an important role in the occurrence and development of liver cancer. In addition, the current study also confirmed that ICOS can regulate PI3K phosphorylation and the expression of AKT.

PI3K is phosphatidyl inositol 3 kinase. PI3K catalyzes phosphatidyl inositol 3 hydroxy phosphorylation. AKT, also known as protein kinase B, is an important kinase located downstream of PI3K. Both PI3K and AKT promote cell survival and maintain normal function. The existing experimental results proved that PI3K and AKT both play an important role in the development of liver cancer cells. Moreover, the activities of PI3K and AKT are up-regulated in liver cancer tissues [21]. Recent studies have shown that the PI3K/AKT/mTOR signaling pathway can be used as the targeting pathway for HCC therapy [24]. Some studies have also been conducted on the expression and significance of PI3K and the associated heat shock protein (HSP) 70 and AKT in liver cells and HCC tissues [21]. Immunohistochemistry, Western blot analysis, and RT-PCR have been used to detect HSP70, PI3K, and AKT protein and mRNA expression in liver cancer tissues and analyzed the association with gender, age, histologic differentiation, lymph node metastasis, and tumor, lymph node, metastasis (TNM) staging, and other clinical pathology parameters. HSP70, PI3K, and AKT protein expression is related to the different degree of liver cancer tissues [25]. Our experimental results also further confirmed the role of P13K in liver cancer cells, and suggested that ICOS may regulate the proliferation and invasion of HepG2 liver cancer cells, and apoptosis through regulating the expression of phosphorylation PI3K and the expression of AKT.

Co-stimulators inhibit the anti-tumor immune response by T and DC cells [17, 18, 20, 22]. In addition, ICOS protects tumor cells from immune escape, and also play an important role in direct attack of antigen-specific cytotoxic T cells at the effector level. Co-stimulatory molecules on the surface of tumor cells induce apoptosis of tumor-specific CTLs and inhibit the immune response to tumors [16]. In contrast, ICOS participates in tumor immune escape, and thus can block the combination of co-stimulatory molecules and their receptors, which cannot only directly inhibit tumor growth, but also suppresses tumor immune escape to further improve activation of initial T cell and CTL killing and improve the immune response against other antigens. Other studies have shown that [16–20] the combination of neutralizing antibodies and ICOS in vitro effectively promotes the production and activity of IFN-γ. At the same time, the activity of cytotoxic T cells with antigen specificity can be enhanced [16, 18, 20, 22, 23]. Therefore, research on ICOS and liver cancer not only provides a new etiologic theory for tumorigenesis, but also provides a new research topic and perspective for diagnosis and treatment of tumors.

## Conclusion

To summarize, because ICOS can restrain the proliferation and invasion of liver cancer cells in vitro, ICOS is expected to become a potential treatment target for liver cancer. Our group will attempt to use ICOS siRNA in liver cancer animal models in the future to observe the curative effect of immune targeted molecular therapy represented by ICOS in vivo.

## Abbreviations

RNAi: RNA interference
ICOS: inducible T-cell co-stimulator
WHO: World Health Organization
HCC: hepatocellular carcinoma
dsRNA: double-stranded RNA
